# Prevalence and risk factors for age-related cataract in Sweden

**DOI:** 10.1080/03009734.2020.1802375

**Published:** 2020-08-06

**Authors:** Magnus Hugosson, Curt Ekström

**Affiliations:** a Department of Ophthalmology, University Hospital, Uppsala, Sweden; b Department of Neuroscience, Ophthalmology, Uppsala University, Uppsala, Sweden

**Keywords:** Cataract, epidemiology, population survey, prevalence, risk factor

## Abstract

**Background:**

Cataract is a major cause of visual impairment worldwide. There is a paucity of prevalence studies from Sweden. Therefore, we report the prevalence of cataract and its risk factors in a population-based study of older adults in Sweden.

**Methods:**

The Tierp Glaucoma Survey was conducted in the municipality of Tierp, Sweden, including 760 subjects aged 65–74 years. The presence of cataract was determined based on retroillumination, with lens opacities evident on slit-lamp examination. To assess risk factors for cataract, odds ratios (ORs) were calculated, adjusted for age and gender.

**Results:**

A total of 234 individuals were found to have cataract, 12 of whom had undergone cataract surgery. The prevalence adjusted for nonparticipation was 31.5% (95% confidence interval [CI] 29.4–33.6), 35.2% (95% CI 28.7–41.8) in females and 26.2% (95% CI 19.8–32.6) in males. Cataract was associated with age ≥70 years (OR 1.93; 95% CI 1.41–2.64), female gender (OR 1.54; 95% CI 1.12–2.11), and myopia (OR 2.3; 95% CI 1.16–3.56), while pseudoexfoliation, smoking, diabetes, hypertension, and ischaemic heart disease were not.

**Conclusion:**

Nearly one-third of the sample were estimated to have lens opacities, or had undergone cataract surgery, making cataract a frequent disorder of older age. The study provided further evidence that increasing age, female gender, and myopia are associated with cataract.

## Introduction

Cataract remains a major cause of visual impairment worldwide (1). However, in many countries cataract surgery is easily available. In fact, cataract surgery is one of the most common surgical procedures performed in Western countries. In Sweden, with a population of 10 million inhabitants, approximately 110,000 cataract surgeries are performed annually according to the Swedish Cataract Register (2). Cataract is a major burden for health-care providers around the world. From a public health perspective, it is important to know the prevalence of undiagnosed lens opacities. Identifying modifiable predictors for the development of cataract will make it possible to initiate strategies to delay the need for cataract surgery. From a societal perspective, it is also important to reduce the costs for health care, especially with an ageing population in mind.

Prevalence studies on cataract and its risk factors have been widely reported from various parts of the world in the past decades. The results of three of these studies on people in the age span of 65–74 years are presented in Table 1. Clearly, in population studies on lens opacities, prevalence rates are a function of the examination methods and diagnostic criteria used. Several classification and grading systems for lens opacities have been developed, including the Wisconsin Cataract Grading System (6) and the Lens Opacities Classification System III (LOCS III) (7). In both systems, photographs were taken of the lenses and were compared to a set of standard colour transparencies of lens opacities. In other grading systems, the findings at biomicroscopy were categorized according to a written description of lens changes (3).

**Table 1.** Prevalence of cataract in subjects 65–74 years of age in the Framingham Eye Study, the Beaver Dam Eye Study, and the Blue Mountains Eye Study.

Study	Ref.	Method	Prevalence (%)
Framingham	(3)	Slit-lamp, ophthalmoscopy	Total 18.0^a^
			Females 19.3
			Males 16.0
Beaver Dam	(4)	Photography	Total 74.3^b^
			Females 76.3
			Males 71.4
Blue Mountains	(5)	Photography	Total 68.4^b^
			Females 73.2
			Males 62.2

^a^ Only individuals with visual acuity of 20/30 or worse in either eye were examined; including subjects with a history of cataract surgery.

^b^ Right eye only.

Previous studies have identified several risk factors associated with cataract, including smoking (8,9), diabetes (10), sunlight exposure (11), high body mass index (12), steroid use (13), increasing age (14,15), female gender (16,17), myopia (17,18), and pseudoexfoliation in the anterior eye segment (19,20). In a study on the Tierp population, pseudoexfoliation was found to be a predictor for cataract surgery (21).

The aim of this investigation was to report the prevalence of age-related cataract and its risk factors in a population survey in Sweden.

## Methods

### The Tierp glaucoma survey

The Tierp Glaucoma Survey was a population-based study on residents in the municipality of Tierp, south-central Sweden, conducted in 1984–86. Its target population comprised 2,429 people, aged 65–74 years. The size of the sample was limited to about one-third of the target population. Of the eligible number of 838 residents, 760 (91%) underwent a detailed eye examination, as described elsewhere (22). The characteristics of participants and non-participants are presented in Table 2.

**Table 2.** Participation in the population survey in Tierp, by age and gender.

	Females (*n* = 429)				Males (*n* = 409)			
	Examined				Examined			
Age	Yes	(%)	No	(%)	Yes	(%)	No	(%)
65 − 69 years	209	(93.3)	15	(6.7)	195	(90.7)	20	(9.3)
70 − 74 years	187	(91.2)	18	(8.8)	169	(87.1)	25	(12.9)
65 − 74 years	396	(92.3)	33	(7.7)	364	(89.0)	45	(11.0)

The study was primarily designed to address the distribution and determinants of open-angle glaucoma. However, a great amount of information was collected, including data on cataract. Briefly, an interview was first held, covering medical and family history. Information was also obtained from medical records. After perimetry, the pupils were dilated to a diameter of at least 3 mm and biomicroscopy undertaken. The presence of cataract was ascertained based on retroillumination using indirect ophthalmoscopy with lens opacities evident on slit-lamp examination. A grading of the amount of opacities in six stages was also performed. Individuals with definite lens opacities in either eye or those who had undergone cataract surgery in one or both eyes were classified as having cataract. The first stage of lens opacities, described as ‘early senile changes’ in the Framingham Eye Study (3), was not accepted as definite cataract in the present study.

The study was approved by the Human Subjects Committee at the Faculty of Medicine, Uppsala University and adhered to the tenets of the Declaration of Helsinki. Informed consent was obtained from all participants.

### Statistical methods

Prevalence estimates were adjusted for non-response by applying the frequency of cataract among those who presented for the examination to those who did not, with age and gender strata. Confidence intervals (CIs) for prevalence rates were calculated using the normal approximation to the binomial distribution. Risk factors for cataract expressed as odds ratios (ORs) were estimated using 2 × 2 tables. Control for age and gender was performed according to Mantel–Haenszel. To simultaneously assess several predictors affecting the risk for cataract, multiple logistic regression analyses were also employed with cataract as the dependent variable.

## Results

### Prevalence of cataract

A total of 234 individuals were found to have cataract, 12 of whom had undergone cataract surgery. The prevalence adjusted for non-response was 31.5% (95% CI 29.4–33.6), 35.2% (95% CI 28.7–41.8) in women and 26.2% (95% CI 19.8–32.6) in men. Another 116 people had early lens changes in either eye, not classified as cataract. The distribution of cataract by age and gender is shown in Table 3. Cataract was strongly related to increasing age in both women and men.

**Table 3.** Prevalence of cataract in the population survey in Tierp, by age and gender.^a^

	Females (*n* = 396)		Males (*n* = 364)	
Age	Prevalence	95% CI	Prevalence	95% CI
65 − 69 years	26.3%	20.3–32.2	22.1%	16.2–27.9
70 − 74 years	45.9%	37.8–52.0	30.8%	23.8–37.7
65 − 74 years^b^	35.2%	28.7–41.8	26.2%	19.8–32.6

Difference (females − males): 9.0% (95% CI −0.2 to 18.1).

^a^ Including past cataract surgery.

^b^ Adjusted for nonparticipation.

CI: confidence interval.

### Prevalence of past cataract surgery

Twelve subjects had a history of cataract surgery, equal to an unadjusted prevalence of 1.6% (95% CI 0.7–2.5). Aphakia in one or both eyes was present in 11 subjects and pseudophakia in either eye in two subjects. In addition, traumatic aphakia was observed in two subjects. Of these, one had age-related cataract in the unaffected eye and was included among the cataract cases.

### Risk factors for cataract

To assess risk factors for cataract, ORs were calculated, adjusted for age and gender. Table 4 provides ORs for potential risk factors. High age, female gender, and myopia increased the risk for cataract, while pseudoexfoliation, current smoking, diabetes mellitus, systemic hypertension, and ischaemic heart disease did not.

**Table 4.** Odds ratios for cataract in the population survey in Tierp, adjusted for age and gender.

		No. of cases		
Characteristics		(*n* = 234)	OR_M-H_	95% CI
Age ≥70 years^a^	No	98	1.00	
	Yes	136	1.93	1.41 − 2.64
Female gender^b^	No	95	1.00	
	Yes	139	1.54	1.12 − 2.11
Pseudoexfoliation, either eye	No	189	1.00	
	Yes	45	1.12	0.75 − 1.68
Myopia, either eye	No	209	1.00	
	Yes	25	2.03	1.16 − 3.56
Current smoker	No	206	1.00	
	Yes	28	0.73	0.46 − 1.17
Diabetes	No	201	1.00	
	Yes	33	1.25	0.79 − 1.99
Hypertension, treated	No	159	1.00	
	Yes	75	1.26	0.90 − 1.78
Ischaemic heart disease	No	195	1.00	
	Yes	39	1.18	0.77 − 1.80

^a^ Adjusted for gender.

^b^ Adjusted for age.

CI: confidence interval; OR_M-H_: Mantel–Haenszel adjusted odds ratio.

The variables mentioned in the previous passage were tested in logistic regression models. The ultimate model included cataract, age as a continuous variable, gender, and myopia. For gender and myopia, the result was almost identical to that of the stratified analyses. Every year of advancing age increased the risk for having cataract with 16% (OR 1.16; 95% CI 1.10–1.23). Females ran a 52% higher risk than males. There was no indication of interactions in the models.

## Discussion

To the best of our knowledge, only one population-based study has previously been reported on cataract prevalence in Sweden, namely the Skövde Cataract Study (23), with the Tierp study being the second. The study in Skövde used the LOCS III grading system, including people 70–84 years of age. A study like the one in Skövde was also conducted in Oulu County, Finland on people aged ≥70 years (24). Furthermore, a case–control study, with controls chosen from the population register, on the association of cataract with diabetes type 2, was undertaken in Laxå, Sweden (25). In this study, a previous version of the LOCS III system was used.

The prevalence of cataract in Tierp was low compared with the population surveys of Beaver Dam, Wisconsin, US and Blue Mountains, Australia (Table 1). However, in subjects 70–74 years of age, the prevalence observed in Tierp was rather close to the prevalence in Oulu (38.2 and 44.6%, respectively). The age-specific prevalence in the Framingham Eye Study was lower than that in Tierp (18.0 and 31.5%, respectively). A likely explanation for the difference can be the more conservative inclusion criteria in the Framingham study; only subjects with a visual acuity of 20/30 or worse were classified as having cataract. In the Laxå study, the prevalence of cataract in the control group of people 65–74 years of age was 72.7% in females and 78.3% in males. Although the estimates were based on only 24 and 36 cases, respectively, the prevalence in Laxå seems to have been much higher than that in Tierp.

The reported prevalence rates of cataract vary for several reasons. Firstly, there is no clear-cut definition of when a lens is opaque enough to be classified as cataract. In the present study, a subject was thought to have cataract if lens opacities were observed on retroillumination using indirect ophthalmoscopy, and the findings were evident on slit-lamp biomicroscopy. It is likely that this method underestimates the frequency of cataract compared with photographic methods. A standardized lens opacity grading system, like LOCS III, should be preferred in population surveys on cataract.

Secondly, the age of the examined population differs between studies. Age is an important risk factor for the development of cataract, as shown in this study. Thirdly, inclusion of subjects with a history of cataract surgery may have an impact on prevalence rates. In the present report, the rates presented for the studies in Framingham, Tierp, Oulu, and Laxå included patients who had undergone surgery, while the rates for Beaver Dam and Blue Mountains did not. Fourthly, an unequal distribution of risk factors for cataract such as exposure to sunlight, smoking habits, and diabetes may affect prevalence rates. Finally, the population survey in Tierp was conducted in 1985–86, while some of the studies referred to in this report were performed more recently. It is not impossible that lifestyle factors have changed over time and thereby modified the prevalence rates.

Consistent with previous research, age was found to be a major risk factor for the development of cataract in both females and males. Accumulated oxidative stress to the lens proteins over the years has been suggested as the primary explanation (26). Furthermore, we confirm findings from previous studies that females are at a higher risk of cataract than males (16,17). It has been suggested that oestrogen plays a role in protecting the lens from oxidative stress (27–29). In this context, decreasing oestrogen levels after menopause would increase the risk for cataract. On the contrary, other studies have concluded that postmenopausal hormone replacement therapy increases the risk for undergoing cataract surgery (30).

The association of age-related cataract with myopia is well-known (17). In most cases, the development of myopia is thought to be a consequence of a shift in refraction in subjects with nuclear lens opacities. However, follow-up studies have demonstrated the possibility of a causal relationship between myopia and cataract, in particular posterior subcapsular cataract (18). With an increasing number of myopic individuals in many countries (31), it is possible that cataract may affect more people and appear earlier in life in the coming years. In the present study, myopia increased the risk for having cataract 2-fold. Unfortunately, adjustment for nuclear opacities could not be performed, which is likely to have biased the result.

Cigarette smoking (8,9) and diabetes (10) are established risk factors for lens opacities. Nevertheless, we were unable to confirm the effect of smoking and diabetes. The sparse number of exposed cases is a likely explanation. Furthermore, we could not verify a relationship between cataract and pseudoexfoliation, reported in other population studies (19,20). Other known risk factors like sunlight exposure (11), high body mass index (12), and steroid use (13) were not examined.

Compared with the population studies in Framingham, Beaver Dam, and Blue Mountains, the Tierp Glaucoma Survey was a small study, limiting its statistical power. However, the high participation rate was a strength of the study. The lack of a standardized grading system of cataract, leading to potential misclassification of disease, was a limitation of the study. Another weakness was that the type of cataract was not registered in the population survey. There are evidence that cortical and nuclear lens opacities do not share the same modifiable risk factors (17,32). To estimate the lifetime risk of cataract surgery in this cohort of older adults, a follow-up study is in progress.

## Conclusion

Nearly one-third of the participants in the Tierp Glaucoma Survey were found to have lens opacities, or had undergone cataract surgery, making cataract a frequent disorder of older age. Nevertheless, the age-specific prevalence was low compared with studies using standardized grading systems based on photography. The study provided further evidence that increasing age, female gender, and myopia are associated with cataract.
